# Relationship between parents’ dietary care and food diversity among preschool children in Japan

**DOI:** 10.1017/S1368980021000963

**Published:** 2022-02

**Authors:** Midori Ishikawa, Kumi Eto, Mayu Haraikawa, Nobuo Yoshiike, Tetsuji Yokoyama

**Affiliations:** 1 Department of Health Promotion, National Institute of Public Health, 2-3-6 Minami, Wako, Saitama 351-0197, Japan; 2 School of Nutrition Sciences, Kagawa Nutrition University, Sakado, Saitama, Japan; 3 Department of Child Studies, Faculty of Child Studies, Seitoku University, Matsudo, Chiba, Japan; 4 Department of Nutrition, Faculty of Health Sciences, Aomori University of Health and Welfare, Hamadate, Aomori, Japan

**Keywords:** Preschool children, Food diversity, Parental care, Nutritional balance, Regular mealtimes

## Abstract

**Objective:**

To identify the relationship between preschool children’s dietary diversity and parents’ care behaviours related to their diet including contents of foods and snacks, mealtime practice and parent–child communication.

**Design:**

Cross-sectional study. Data were extracted from the National Nutrition Survey on Preschool Children in 2015 by Japan’s Ministry of Health, Labour and Welfare.

**Setting:**

The distribution of food diversity score (FDS) (maximum of eight points) was confirmed. The participants were divided into higher (≥4 points) and lower (≤3 points) food diversity groups. A comparison between the two groups examined parents’ socio-economic status, children’s health and living conditions, and parental care concerning children’s diets (thirteen items). A multiple regression analysis was performed relating FDS to the factors of parental socio-economic status and child health, and a logistic regression analysis was conducted to identify factors of parental care related to the higher food diversity group.

**Participants:**

2143 persons from households with children aged 2–6 years.

**Results:**

Parental care concerning children’s diets was the factor most strongly associated with children’s FDS. Those factors most strongly associated with higher food diversity were nutritional balance of foods (OR: 1·76; 95 % CI 1·44, 2·16; *P* < 0 0001), snack contents (OR: 1·41; 95 % CI 1·07, 1·86; *P* = 0·014) and regular mealtimes (OR: 1·30; 95 % CI 1·08, 1·55; *P* = 0·005).

**Conclusions:**

The findings indicate the importance of parents paying attention to the contents of children’s foods and snacks, ensuring that children eat regularly, and increasing the diversity of their diets.

Eating a variety of foods in early childhood is particularly well recognised as being important for optimal nutritional status across the life course^(1–3)^. According to the United Nations FAO, food variety refers to the consumption of a mixture of foods from a range of food groups^(4)^. The FAO global dietary guidelines recommend that people should eat a wide variety of food for a balanced diet and, as such, introduced the food diversity score (FDS). The FDS assesses the diet of people at the local level, whereby diversity in the number of food groups can be used as an indicator to assess the nutritional quality of the whole diet and has been promoting the assessment of household and individual dietary diversity worldwide^(4,5)^.

In previous studies, a dietary plan with practical food amount based on a variety of foods within preschool children’s energy requirements was proposed^(6,7)^. However, many young children develop unbalanced diets due to picky eating, among other habits^(8,9)^. The National Nutrition Survey on Preschool Children (NNSPC) conducted in Japan reported that approximately 80 % of parents expressed frequent concerns about the dietary habits of their children^(10)^. Studies have particularly linked limited dietary variety to low intakes of fruits and vegetables and high intakes of unhealthy processed food^(11,12)^, with possible consequences including obesity^(12)^.

Preschool children’s dietary behaviours and diet quality are associated with home environment and parental behaviours^(13,14)^. Many parents recognise the need to be aware of and closely manage their children’s diet to ensure food diversity, including contents of foods and snacks (e.g., nutritional balance, flavouring and seasoning, and amount of food), mealtime practice (e.g., regular mealtimes and chewing well) and parent–child communication (e.g., cooking meals with children and eating together)^(10)^. Previous research has related parents and children cooking meals together to higher food diversity of children’s diets^(15)^. However, few academic studies have comprehensively examined how parental care behaviours are related to children’s food diversity.

Therefore, the aim of the current study was to identify the relationship between preschool children’s food diversity and their parents’ care behaviours with regard to their diet including contents of foods and snacks, mealtime practice and parent–child communication.

## Methods

Data for the study were retrieved from the NNSPC, which was conducted on September 2015 by the Ministry of Health, Labour and Welfare (MHLW) in Japan^(10)^. The NNSPC aims to obtain basic data for promoting breast-feeding and improving the eating habits of infants and preschool children by understanding the actual conditions of nutrition and diet of infants and preschoolers across the nation. This survey is conducted every 10 years. The survey method and items were examined by establishing an expert study group in the MHLW.

### Study population and procedure

**Fig. 1:**
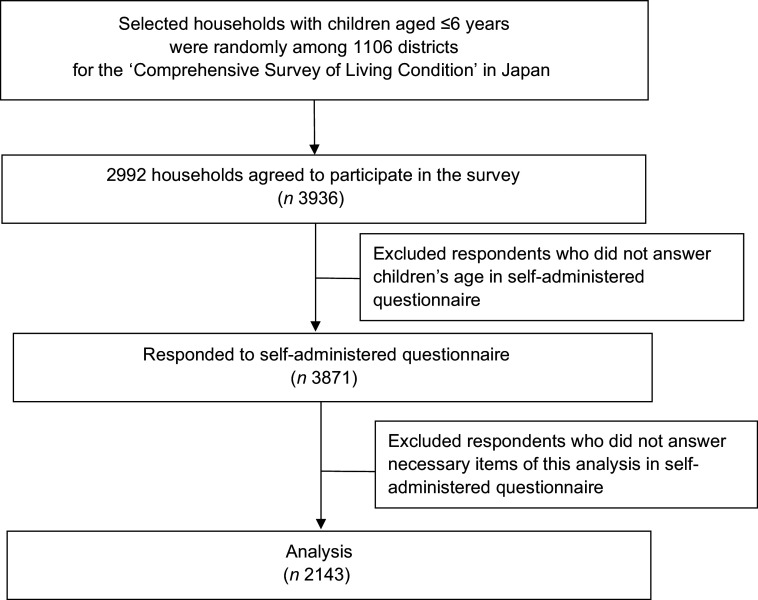
Study population and procedure diagram of the current study

Figure 1 shows the study population and procedure. Children aged ≤6 years as of 31 May 2015 were randomly selected from households among 1106 districts for the Comprehensive Survey of Living Conditions, conducted by the MHLW. Three districts affected by heavy rain in September 2015 were excluded from the survey target area. First, the MHLW explained the survey method to the prefectures. Subsequently, the prefectural public health centre employed investigators to visit the households selected for this survey. The investigators asked the children’s mothers (or caregivers) to complete a questionnaire, which was collected at a later date. In total, 2992 households with 3936 children aged ≤6 years participated in the survey. The response rate of the survey was 56·8 %. The questionnaires associated with sixty-five children were excluded because information on age was not available. Finally, 3871 questionnaires were collected for analysis^(10)^. A database was prepared by the Maternal and Child Health Division, Department of Equal Employment and Children’s Family, MHLW.

The NNSPC has two types of questionnaires, one of which is restricted to infants aged <2 years and the other of which encompasses children aged 2–6 years. Data obtained from the latter questionnaire were used in the current study. In total, 2143 persons responded to all items consistent with the purpose of the current study.

### Measurement

Children’s dietary patterns in relation to the eight food groups (grains, fish, meat, eggs, soyabeans/soya products, vegetables, fruit and milk) were evaluated as objective variables, as well as their intake of processed foods, whereby four items (sweetened beverages, confectioneries, instant noodles and fast food) were investigated. The survey inquired how often the children consumed foods in each group (≥2 times/d, once a day, 4–6 d/week, 1–3 d/week or less than once a week or rarely)^(10,16)^. The FAO’s FDS was applied to assess the nutritional quality of the whole diet^(5)^.

Thirteen items assessed parents’ care behaviour in relation to children’s diets based on previous studies, which confirm their validity and reliability, with professional support to provide nutrition consultation. We posed the question ‘Are you (parent) careful about your child’s diet?’ with regard to the following thirteen items: (1) food : seven items comprising nutritional balance, flavouring and seasoning, size or softness, assorted arrangements and colours (colour and placement of the cooked foods on the plate), amount and snack (contents, amount)^(17–20)^; (2) mealtime practice: three items comprising regular mealtimes, chewing well, table manners^(17,21)^ and (3) parent–child communication: three items comprising enjoyment of eating, eating together, cooking together^(17,18)^. Of the thirteen items related to parental care emphasised in the current study, five items (nutrition balance, amount, regular mealtimes, table manners and eating together) were researched in both the 2005 and 2015 surveys, and eight other items were newly added in the 2015 survey. Each item was scored based on ‘yes’ or ‘no’ responses.

Explanatory variables related to parents included their relationships with their children, age of mother, current employment status of mother, household structure (i.e., presence of other children, grandparents and others), subjective economic status, leisure time (i.e., affluent, somewhat, neither, not so much, unable to afford at all), the place where the child spends time during the day (i.e., nursery school, kindergarten, centres for early childhood education and care, grandparents and relatives, or none of the above) and lifestyle regarding eating breakfast with parents. In addition, data concerning children’s age; height; weight; nutritional status (degree of obesity); food allergies; tooth decay and time spent on TV, video and games were obtained.

### Nutritional status of children

The nutritional status of children was determined based on body weight and height. The degree of obesity (%) was calculated using the following formula: self-reported body weight (g) − standard body weight (g) for height/standard body weight (g) for height × 100. The judgement criteria for the degree of obesity were ‘obese’ (≥30 %), ‘overweight’ (20–30 %), ‘tendency to be overweight’ (15–20 %), ‘standard’ (−15 to +15 %), ‘tendency to be underweight’ (< 15 % to < 20 %) and ‘underweight’ (< 20 %). The standard body weight was calculated using the formula of standard body weight for height in Japanese children^(22,23)^. The formula does not consider age because the standard body weight for height curves was almost identical for children aged 1–6 years^(24)^.

Height and weight were self-reported questions because there is a rule that the same item should not be surveyed repeatedly to the people by different surveys according to the regulations of the MHLW. As the height and weight of children are surveyed by the National Growth Survey on Preschool Children in 2010^(24)^, these data were not measured in the NNSPC and were asked in a self-reported method. However, in Japan, many parents measure the physique of an infant or preschool child at home; it is also often measured at day care centres and kindergartens. Therefore, it might be considered that several measured values were described in the survey.

### Statistical analysis

The FDS of children comprised the total number of eight food groups being consumed at least once a day^(4,10,19)^. The FDS was one point if consumption occurred once or more per day or zero points if less than that. There were eight types of foods; thus, the maximum score was eight points. Once the FDS distribution was identified, the FDS was divided into two groups according to medians: (1) three or fewer points and (2) four or more points^(4)^. The processed food score was calculated according to the total number of four food items (sweetened beverages, confectioneries, instant noodles and fast food) being consumed at least once a day^(4)^. As in the case of FDS, the processed food score was calculated as a score of one point if the food type was consumed at least once a day or zero points if less than that. There were four types of foods; thus, the maximum score was four points.

The sex of the parent who answered the questionnaire, age and socio-economic status as well as children’s sex; nutritional status; food allergies; tooth decay and time spent on TV, video and games were compared between the two FDS groups. Furthermore, the total value calculated from the thirteen items of the parent’s care behaviour in children’s diets was compared between the two FDS groups.

Multiple regression analysis analysed relationships between FDS scores and several variables, including the total value of the parent’s care behaviours in children’s diets; subjective socio-economic status; food allergies; tooth decay and time spent on TV, video and games, as well as the child’s age and mother’s age. The continuous variables included the total value of the parent’s care behaviours in children’s diets, the child’s age and the mother’s age. The nominal variables such as subjective socio-economic status; food allergies; tooth decay and time spent on TV, video and games were converted to an ordinal scale.

Next, we used logistic regression to specifically analyse the relationship between the variables and the higher FDS group. Multivariate analysis was performed for each of the thirteen items measuring parental care in relation to children’s diets using a logistic regression model that adjusted for the parent’s relationship with the child, child’s sex, employment status of the mother and household structure (model 1).

Additional multivariate analysis was performed for each of the thirteen items measuring parental care in relation to children’s diets using a logistic regression model that adjusted for the parent’s relationship with the child, child’s sex, employment status of the mother, household structure, subjective economic status, leisure time and place where the child spends time during the day (model 2).

All statistical analyses were performed using SAS software, version 9·4 (SAS Institute, Inc.). A probability (*P*) value of <0·05 was considered statistically significant.

## Results

**Fig. 2:**
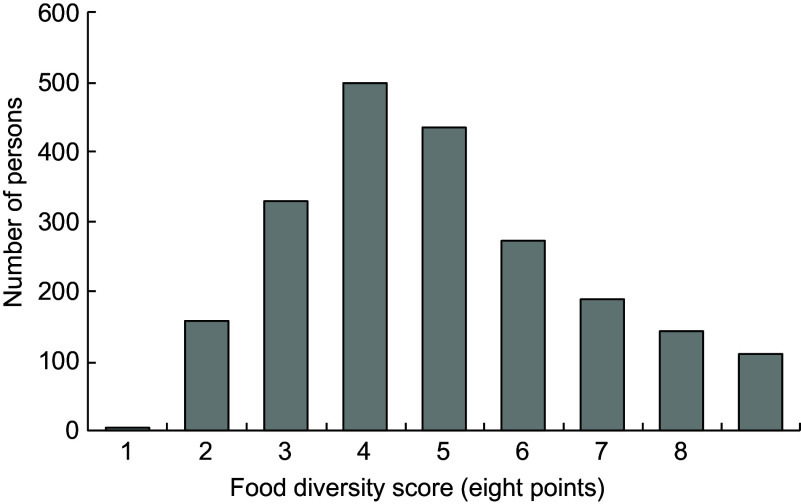
Distribution of food diversity score in children

Figure 2 shows the children’s FDS distribution. Scores ranged from 0 to 8 points, and there was a normal distribution with a median of four points. The median was divided into two groups: ≥4 points (*n* 1151) and ≤3 points (*n* 992).

Table 1 compares the characteristics of mother’s age (*P* = 0·001) and employment status, family members living together, subjective economic status and leisure time, and the places where children spend the day according to FDS group. The mean age of mother (36·3 years) in the ‘higher FDS’ group was 1 year older than those in the ‘lower FDG’ group (35·5 years) (*P* = 0·001). The subjective economic status of the ‘higher FDS’ group was better than that of the ‘lower FDS’ group (*P* < 0·0001). In addition, children of the higher FDS group were more likely to be in nursery school (*P* = 0·041), whereas children of the lower FDS group were more commonly in kindergarten (*P* = 0·034). A higher proportion of parents in the lower FDS group tended to skip breakfast (*P* = 0·0002). There were no significant differences in the other variables between the two groups.

**Table 1:** Parents’ socio-economic status by food diversity group*

			Food diversity score group				*P*
			Higher (≥4 points)		Lower (≤3 points)		
			(*n* 1151, 53·7 %)		(*n* 992, 46·3 %)		
			*n*	%	*n*	%	
Relationship with their child		Mother	1130	98·2	968	97·6	0·338
		Father	21	1·8	24	2·4	
Age of mother (years old)†		Mean	36·3		35·5		0·001
		sd		5·0		5·3	
Employment status of mother	Currently work	Yes	654	56·8	558	56·3	0·791
		No	497	43·2	434	43·7	
Household structure (whether living together or not)	Single	Mother or father and one child	40	3·5	46	4·6	0·439
		Mother or father and grandparent and one child	40	3·5	25	2·5	
	Two generations	Mother and father and one child	192	16·7	150	15·1	
		Mother and father and children	684	59·3	599	60·5	
	Three generations	Mother and father and grandparent and children	194	16·9	170	17·1	
	Others	Others (living together with non-family adults)	1	0·1	2	0·2	
Subjective economic status		Affluent	98	8·5	79	8·0	<0·0001
		Somewhat	282	24·5	166	16·7	
		Neither	379	32·9	328	33·1	
		Not so much	299	26·0	325	32·7	
		Unable to afford at all	93	8·1	93	9·4	
		Do not want answer	0	0·0	1	0·1	
Leisure time		Affluent	93	8·1	81	8·2	0·398
		Somewhat	274	23·8	216	21·8	
		Neither	244	21·2	233	23·5	
		Not so much	416	36·1	371	37·3	
		Unable to afford at all	124	10·8	90	9·1	
		Do not want answer	0	0·0	1	0·1	
Place where the child spends time during the day	Nursery school	Yes	491	42·7	380	38·3	0·041
		No	660	57·3	612	61·7	
	Kindergarten	Yes	415	36·1	402	40·5	0·034
		No	736	63·9	590	59·5	
	Centres for early childhood education and care	Yes	77	6·7	64	6·5	0·825
		No	1074	93·3	928	93·5	
	Grandparents and relatives	Yes	49	4·3	58	5·9	0·092
		No	1102	95·7	934	94·1	
	None of the above	Yes	142	12·3	123	12·4	0·965
		No	1109	87·7	869	87·6	
Lifestyle	Eating breakfast	Everyday	1103	95·8	910	91·7	0·0002
		4–5 d/week	39	3·4	68	6·9	
		2–3 d/week	4	0·4	1	0·1	
		1 d or less per week	5	0·4	13	1·3	
		I do not eat at all	0	0·0	0	0·0	

* *χ*^2^ test.

† *t* test.

Table 2 compares the children’s age; sex; nutritional status; food allergies; tooth decay and time spent on TV, video or games between the two FDS groups. The nutritional status did not point to a significant relationship with FDS. In total, 92·1 and 93·6 % of children in the higher and lower FDS groups, respectively, were included in the standard range for Japanese children. The children in the lower FDS group had more tooth decay (*P* = 0·006), whereas those in the higher FDS group had spent < 2 h/d on TV, video or games during the weekdays (*P* = 0·005) and weekends (*P* = 0·002).

**Table 2:** Child health and lifestyle situation by food diversity group*

			Food diversity score group				*P*
			Higher (≥4 points)		Lower (≤3 points)		
			(*n* 1151, 53·7 %)		(*n* 992, 46·3 %)		
			*n*	%	*n*	%	
Age†	Years old						0·208
	Mean		4·2		4·3		
	sd			1·1		1·1	
Sex	Male		582	50·6	522	52·6	0·342
	Female		569	49·4	470	47·4	
Nutritional status‡	Height (cm)		100·8	8·6	100·9	8·3	0·836
	Weight (kg)		15·8	2·9	15·9	2·8	0·550
	+30% ≤ (obesity)		4	0·4	10	1·0	0·151
	+30 to 20% (overweight)		14	1·2	12	1·2	
	+15 to 20% (overweight tendency)		36	3·1	21	2·1	
	−15 to + 15% (standard)		1060	92·1	928	93·6	
	−15 to −20% (underweight tendency)		25	2·2	14	1·4	
	≤−20% (underweight)		12	1·0	7	0·7	
Food allergy symptoms		Yes	184	16·0	162	16·3	0·829
		No	967	84·0	880	83·7	
Tooth decay		Yes	194	16·9	214	21·6	0·006
		No	955	83·1	777	78·4	
Time spent on TV, video or games							
	Weekday	None	16	1·4	13	1·3	0·005
		<2 h/d	908	78·9	725	73·1	
		≥2 h/d	227	19·7	254	25·6	
	Weekend	None	10	0·9	9	0·9	0·002
		<2 h/d	704	61·1	533	53·7	
		≥2 h/d	437	38·0	450	45·4	

* *χ*^2^ test.

† : *t* test.

‡ The standard body weight for height in Japanese children.

Table 3 presents differences in food intake between two FDS groups. The higher FDS group had higher frequencies of grains, fish, meat, eggs, soyabeans/soya products, vegetables, fruits and milk intake than the lower FDS group but less frequencies of instant noodle and fast-food intake.

**Table 3:** Food intakes by food diversity group

	Food category	Frequency	Food diversity score group				P*
			Higher (≥4 points)		Lower (≤3 points)		
			(*n* 1151, 53·7 %)		(*n* 992, 46·3 %)		
			*n*	%	*n*	%	
Food group	Grain	≥ 2 times per day	1137	98·8	958	96·6	0·002
		Once a day	12	1·0	21	2·1	
		4–6 d/week	1	0·1	11	1·1	
		1–3 d/week	0	0	2	0·2	
		Less than once a week	1	0·1	0	0·0	
		Have not eaten yet	0	0	0	0·0	
	Fish	≥ 2 times per day	116	10·1	4	0·4	<0·0001
		Once a day	244	21·2	10	1·0	
		4–6 d/week	290	25·2	213	21·5	
		1–3 d/week	460	39·9	682	68·7	
		Less than once a week	40	3·5	81	8·2	
		Have not eaten yet	1	0·1	2	0·2	
	Meat	≥ 2 times/d	264	22·9	16	1·6	<0·0001
		Once a day	386	33·5	48	4·8	
		4–6 d/week	365	31·7	583	58·8	
		1–3 d/week	132	11·5	325	32·8	
		Less than once a week	3	0·3	18	1·8	
		Have not eaten yet	1	0·1	2	0·2	
	Eggs	≥ 2 times/d	85	7·4	4	0·4	<0·0001
		Once a day	430	37·3	48	4·8	
		4–6 d/week	336	29·2	425	42·9	
		1–3 d/week	230	20·0	407	41·0	
		Less than once a week	54	4·7	93	9·4	
		Have not eaten yet	16	1·4	15	1·5	
	Soyabeans and soya products	≥ 2 times/d	150	13·0	6	0·6	<0·0001
		Once a day	417	36·2	31	3·1	
		4–6 d/week	307	26·7	363	36·6	
		1–3 d/week	240	20·9	488	49·2	
		Less than once a week	36	3·1	100	10·1	
		Have not eaten yet	1	0·1	4	0·4	
	Vegetables	≥ 2 times per day	836	72·7	331	33·4	<0·0001
		Once a day	273	23·7	241	24·3	
		4–6 d/week	28	2·4	271	27·3	
		1–3 d/week	13	1·1	125	12·6	
		Less than once a week	1	0·1	21	2·1	
		Have not eaten yet	0	0·0	3	0·3	
	Fruit	≥ 2 times/d	221	19·2	12	1·2	<0·0001
		Once a day	521	45·2	66	6·7	
		4–6 d/week	216	18·8	379	38·2	
		1–3 d/week	154	13·4	398	40·1	
		Less than once a week	38	3·3	132	13·3	
		Have not eaten yet	1	0·1	5	0·5	
	Milk	≥ 2 times/d	533	46·3	237	23·9	<0·0001
		Once a day	499	43·4	285	28·8	
		4–6 d/week	52	4·5	263	26·5	
		1–3 d/week	45	3·9	164	16·5	
		Less than once a week	10	0·9	36	3·6	
		Have not eaten yet	12	1·0	7	0·7	
Processed food	Sweetened beverage	≥ 2 times/d	125	10·9	95	9·6	0·491
		Once a day	239	20·8	198	19·9	
		4–6 d/week	165	14·3	160	16·1	
		1–3 d/week	370	32·1	339	34·2	
		Less than once a week	224	19·5	183	18·5	
		Have not eaten yet	28	2·4	17	1·7	
	Confectionery	≥ 2 times /d	149	13·0	108	10·9	0·212
		Once a day	555	48·1	483	48·8	
		4–6 d/week	198	17·2	189	19·0	
		1–3 d/week	177	15·4	167	16·8	
		Less than once a week	64	5·6	42	4·2	
		Have not eaten yet	8	0·7	3	0·3	
	Instant noodle	≥ 2 times/d	0	0	0	0	<0·0001
		Once a day	2	0·2	3	0·3	
		4–6 d/week	9	0·8	5	0·5	
		1–3 d/week	77	6·7	121	12·2	
		Less than once a week	804	69·8	738	74·4	
		Have not eaten yet	259	22·5	125	12·6	
	Fast food	≥ 2 times/d	0	0·0	0	0·0	0·004
		Once a day	4	0·4	1	0·1	
		4–6 d/week	11	1·0	7	0·7	
		1–3 d/week	110	9·5	126	12·7	
		Less than once a week	941	81·7	816	82·3	
		Have not eaten yet	85	7·4	42	4·2	
			lsmean	se	lsmean	se	
Food diversity score†		8 points/d	5·3	1·3	2·3	0·8	0·0002
Processed food score‡		4 points/d	0·9	0·8	0·9	0·8	<0·0001

* *χ*^2^ test.

† ANCOVA. Food diversity score: the total number of eight food groups (grain, fish, meat, eggs, soyabeans and soya products, vegetables, fruit and milk) eaten at least once a day.

‡ Adjusted relationship for the child, sex of child, employment status of mother, family living together, subjective economic status and leisure time, caregiver of the child during the day. Processed food score: the total number of four food items (sweetened beverage, confectionery, instant noodle and fast food) eaten at least once a day.

Table 4 compares the proportion of parental care in relation to children’s diets (thirteen items) between the two FDS groups. In the higher FDS group, the proportion of parents who reported being careful with respect to nutritional balance (*P* < 0·001), flavouring and seasoning (*P* = 0·004), assorted arrangement and colours (*P* = 0·002), contents of snack (*P* < 0·001), amount of snack (*P* = 0·015), regular mealtimes (*P* < 0·001), chewing well (*P* = 0·002), enjoying eating (*P* = 0·032) and eating together (*P* = 0·030) were significantly higher than in the lower FDS group.

**Table 4:** Parent’s care on diet of child by food diversity group

			Food diversity score group				P*
			Higher (≥4 points)		Lower (≤3 points)		
			(*n* 1151, 53·7 %)		(*n* 992, 46·3 %)		
			*n*	%	*n*	%	
Parent’s care on diet of child (thirteen items)								
Food	Nutritional balance	Yes	923	80·2	662	66·7	<0·0001
		No	228	19·8	330	33·3	
	Flavouring and seasoning	Yes	477	41·4	351	35·4	0·004
		No	674	58·6	641	64·6	
	Size or softness	Yes	248	21·6	191	19·3	0·190
		No	903	78·4	801	80·7	
	Assorted arrangements and colours	Yes	253	22·0	164	16·5	0·002
		No	898	78·0	828	83·5	
	Amount	Yes	569	49·4	452	45·6	0·074
		No	582	50·6	540	54·4	
Snack	Contents	Yes	189	16·4	98	9·9	<0·0001
		No	962	83·6	894	90·1	
	Amount	Yes	464	40·3	349	35·2	0·015
		No	687	59·7	643	64·8	
Mealtime practice	Regular mealtimes	Yes	576	50·0	399	40·2	<0·0001
		No	575	50·0	593	59·8	
	Chewing well	Yes	356	30·9	246	24·8	0·002
		No	795	69·1	746	75·2	
	Table manners	Yes	794	69·0	660	66·5	0·226
		No	357	31·0	332	33·5	
Parent–child communication	Enjoyment of eating	Yes	592	51·4	464	46·8	0·032
		No	559	48·6	528	53·2	
	Eating together	Yes	826	71·8	669	67·4	0·030
		No	325	28·2	323	32·6	
	Cooking together	Yes	134	11·6	96	9·7	0·143
		No	1017	88·4	896	90·3	
			Mean	sd	Mean	sd	*P*
Total number of the parent’s care on diet of child (thirteen points)†			5·6	2·7	4·8	2·6	<0·0001

* *χ*^2^ test.

† *t* test.

Table 5 shows the results of the multiple regression analysis of factors related to FDS. Parental care behaviours concerning children’s diets were strongly associated with children’s FDS. The total number of the items of parental care of children’s diets (*P* < 0·001) and mother’s age (*P* = 0·01) were positively associated with FDS, whereas subjective economic status (*P* = 0·003) and TV, video or games during the weekdays (*P* = 0·01) were negatively associated with FDS.

**Table 5:** Factors related to food diversity score (*n* 2143)*,†,‡

Factors	Standardised parameter estimate	*P*
Total number of the parental care on diet of child	0·16	<0·0001
Age of mother	0·06	0·01
Age of child	−0·04	0·08
Subjective economic status	−0·06	0·003
Time spent on TV, video or games (weekday)	−0·06	0·01
Skipping breakfast of mother	–	
Food allergy symptoms	–	
Tooth decay	–	

*P*: selected by stepwise method.

* Factors related to FDS: total number of the parent’s care on diet of children, subjective economic status, food allergies, tooth decay, time spent on TV, video or game, child’s age and mother’s age.

† Continuous variable: total number of the parent’s care on diet of children, child’s age and mother’s age.

‡ Nominal variable (ordinal scale): subjective economic status (1: affluent, somewhat, 2: neither, 3: not so much, unable to afford at all, do not want answer), food allergies symptoms (1: yes, 2: no), tooth decay, (1: yes, 2: no) and time spent on TV, video or game (1: none, 2:< 2 h/d, 3:≥ 2 h/d).

Table 6 shows the results of the associations between ‘parental care of children’s diets’ and ‘FDS group’ using stepwise multivariate analysis.

**Table 6:** Relationship between food diversity and parent’s care on diet of child (*n* 2143)*

			Model 1†			Model 2‡			Stepwise		
			OR	95 % CI	*P*	OR	95 % CI	*P*	OR	95 % CI	*P*
Parent’s care on diet of child (thirteen items)											
Food	Nutritional balance	Yes	1·91	1·56, 2·35	<0·0001	1·91	1·56, 2·35	<0·0001	1·76	1·44, 2·16	<0·0001
		No	1·00			1·00			1·00		
	Flavouring and seasoning	Yes	1·24	1·03, 1·48	0·022	1·24	1·03, 1·48	0·020			
		No	1·00			1·00					
	Size or softness	Yes	1·17	0·94, 1·45	0·160	1·17	0·94, 1·45	0·163			
		No	1·00			1·00					
	Assorted arrangements and colours	Yes	1·35	1·08, 1·69	0·009	1·34	1·07, 1·68	0·011			
		No	1·00			1·00					
	Amount	Yes	1·18	0·99, 1·41	0·061	1·19	1·00, 1·41	0·057			
		No	1·00			1·00					
Snack	Contents	Yes	1·72	1·32, 2·25	<0·0001	1·72	1·31, 2·24	<0·0001	1·41	1·07, 1·86	0·014
		No	1·00			1·00			1·00		
	Amount	Yes	1·23	1·03, 1·48	0·026	1·23	1·03, 1·48	0·027			
		No	1·00			1·00					
Mealtime practice	Regular mealtimes	Yes	1·45	1·21, 1·73	<0·0001	1·44	1·21, 1·72	<0·0001	1·30	1·08, 1·55	0·005
		No	1·00			1·00			1·00		
	Chewing well	Yes	1·34	1·10, 1·63	0·003	1·34	1·10, 1·63	0·004	1·20	0·98, 1·46	0·076
		No	1·00			1·00			1·00		
	Dietary manners	Yes	1·11	0·92, 1·34	0·273	1·11	0·92, 1·34	0·282			
		No	1·00			1·00					
Parent–child communication	Enjoyment of eating	Yes	1·16	0·97, 1·38	0·097	1·16	0·97, 1·38	0·100			
		No	1·00			1·00					
	Eating together	Yes	1·23	1·02, 1·48	0·034	1·22	1·01, 1·48	0·037			
		No	1·00			1·00					
	Cooking together	Yes	1·20	0·90, 1·60	0·216	1·19	0·89, 1·58	0·246			
		No	1·00			1·00					

* Food diversity score (0: ≤ 3 points; 1: ≥ 4 points).

† Model 1: adjusted for the relationship with the child (mother or father), child’s sex, employment status of parent (yes or no) and family members in the household (other children, grandparents and others).

‡ Model 2: adjusted for the relationship with the child (mother or father), child’s sex, employment status of parent (yes or no), family living together (other children, grandparents and others), subjective economic status (affluent, somewhat, neither, not so much or unable to afford at all), leisure time (affluent, somewhat, neither, not so much, unable to afford at all) and place where the child spends time during the day (nursery school, kindergarten, centre for early childhood education and care, with grandparents, with relatives, staying at home).

A number of model 1 variables were identified as predictors for FDS. Five of seven factors in the food category were positively and significantly associated with FDS: nutritional balance (OR = 1·91; 95 % CI 1·56, 2·35; *P* < 0·001); flavouring and seasoning (OR = 1·24; 95 % CI 1·03, 1·48; *P* = 0·022) and assorted arrange and colours (OR = 1·35; 95 % CI 1·08, 1·69; *P* = 0·009). The content and amount of snack category were positively and significantly associated with FDS; contents (OR = 1·72; 95 % CI 1·32, 2·25; *P* < 0·001) and amount (OR = 1·23; 95 % CI 1·03, 1·48; *P* = 0·026). Two of the three factors in the ‘mealtime practice’ category were positively associated with FDS, namely regular mealtimes (OR = 1·45; 95 % CI 1·21, 1·73; *P* < .0·001) and chewing well (OR = 1·34; 95 % CI 1·10, 1·63; *P* = 0·003). With regard to the parent–child communication category, only eating together (OR = 1·23; 95 % CI 1·02, 1·48; *P* = 0·034) was significantly associated with FDS.

The model 2 analysis confirmed the variables associated with FDS. For ‘food’, the same results as those identified for model 1 above were achieved for nutritional balance (OR = 1·91; 95 % CI 1·56, 2·35; *P* < 0 0001) and flavouring and seasoning (OR = 1·24; 95 % CI 1·03, 1·48; *P* = 0·020). However, slightly different yet still significant results were found related to assorted arrangements and colours (OR = 1·34; 95 % CI 1·07, 1·68; *P* = 0·011). Furthermore, snack contents and snack amounts were associated with FDS (OR = 1·72; 95 % CI 1·31, 2·24; *P* < 0·001; OR = 1·23; 95 % CI 1·03, 1·48; *P* = 0·027, respectively). Similarly, for the ‘mealtime practice’ category, the same results were identified for chewing well (OR = 1·34; 95 % CI 1·10, 1·63; *P* = 0·004); however, slightly different values were found regarding the association between FDS and regular mealtimes (OR = 1·44; 95 % CI 1·21, 1·72; *P* < 0 0·001). In the parent–child communication category, eating together (OR = 1·22; 95 % CI 1·01, 1·48; *P* = 0·037) was again the only factor significantly associated with FDS.

The results of the stepwise analysis identified several predictors for FDS, including nutritional balance of food (OR = 1·76; 95 % CI 1·44, 2·16; *P* < 0·001), snack contents (OR = 1·41; 95 % CI 1·07, 1·86; *P* = 0·0014) and regular mealtimes (OR = 1·30; 95 % CI 1·08, 1·55; *P* = 0·005).

## Discussion

The current study identified that lower food diversity was associated with a higher likelihood that parents skipped breakfast and greater consumption of processed or fast foods, as well as more time spent per day on TV, video or games. Furthermore, the higher FDS group was associated with greater parental care about the contents of children’s diets and qualitative aspects of eating, such as regular mealtimes and eating together

In Japan, where the social trend of people owning and spending time on smart phones or tablet PC has been increasing, it has been reported that a mother’s unhealthy lifestyle correlates strongly with prolonged screen time among school-aged children^(25)^. A European longitudinal study targeted 2–9-year-old children and indicated the effects of TV viewing and other screen activities for young children, both on their consumption of sugary drinks and an increase in BMI^(26)^. In Japan as well, it would be necessary to study the effect of screen time during early childhood on the children’s food and snack intake.

In Japan’s NNSPC, the proportion of parental participants who ensured the nutritional balance of foods (72·0 %) was higher than those who were careful about snack contents (12·4 %) and having regular mealtimes (45·0 %). The effects of meal timing and frequency on children’s health have been a research topic for many years, and changing the behaviour of parents and children who do not have regular mealtimes is a complex issue. Previous study findings have indicated a close relationship between children’s mealtimes and their parents’ working times and other lifestyle elements^(27)^.

It may be possible to change parents’ behaviour and increase dietary diversity by providing nutritional guidance on the contents of foods and snacks consumed both in and outside out of the home^(28)^. In addition, to combat picky eating habits, it is important to promote interventions that support skills for food choice and preparation^(29)^ and food environments that change the diet quality at home^(15,30,31)^. According to Helland *et al*., behaviours that can improve food diversity in early childhood include modelling, responsive feeding, repeated exposure and enjoyable meals^(11)^.

According to the results of the NNSPC, 41·9 % of 2–3-year-old and 28·9 % of 5-year-old and above children consume sweetened beverages and confectionaries twice a day or more, as snacks. The proportion of those who did not have a fixed snack time was 43·7 %^(10)^. As such, early childhood nutritional education should include information on both snack contents and timing.

In addition, to broaden the food diversity of children, it is important to consider the content of snacks. Prior research has identified fruits, milk and dairy products among the top ten most frequently eaten foods and beverages consumed as snacks by children in Australia, China, Mexico and the USA; however, confectionery, cookies, candy, ice creams and cakes are also seen among the top ten snacks. These undermine the nutritional benefits of healthier foods and contribute to poor dental health^(19)^. Some dental investigations of preschool children suggest a significant relationship between snack items (e.g., sweet buns) and caries^(32–34)^.

Evidence of the effects of snacks on health status, especially in children, is still weak. The lack of consistent evidence related to this issue may be partly due to a non-standardised definition of snack contents^(35)^. In the future, researchers should investigate snack contents and amounts within the early childhood population in Japan to inform public policy for healthy snacks in this age group.

There were several limitations to the current study that should be addressed. First, the response rate of the survey was only 56.8 %. We relied on the 2015 database of the NNSPC conducted by the MHLW. In that investigation, 3871 questionnaires were collected from 3936 children; however, only 2143 participants responded to all of the survey items. The most unanswered items concerned height, weight and subjective economic status. It may have been difficult for some parents to subjectively gauge their economic status. However, the height and weight might be measurable at homes, daycare centres or kindergartens.

Second, the items related to the effects of the food habits of parents on the food diversity of children are limited in the NNSPC. Parental habits may influence the food diversity of the children and parents^(13,14,17,21,31)^. Furthermore, information regarding socio-economic status was self-reported. Analysing the actual situation through socio-economic status is necessary for the survey rather than relying on self-reported information. In the future, therefore, cross-referencing the data from the NNSPC with those of other national surveys that measure actual socio-economic status (e.g., Comprehensive Survey of Living Conditions) may be necessary for analysis.

## Conclusion

The current study assessed relationships between young children’s dietary diversity and parental care behaviours regarding foods and found that parental care was a predictor of greater food diversity. Children’s diets are strongly based on parents’ care concerning the contents of children’s foods and snacks and regular mealtimes. The results of the current study can be used to inform efforts to develop and implement nutritional guidance education for parents and nutrition staff, including school meal providers.

### Availability of data and materials

Permission for the use of the dataset in the current study was obtained from the MHLW, Japan. All data belong to the MHLW, and the database cannot be used for other studies.
